# Romiplostim Reduces Platelet Transfusion Needs in Multiple Myeloma Patients Undergoing Autologous Stem Cell Transplantation

**DOI:** 10.7759/cureus.76164

**Published:** 2024-12-21

**Authors:** Vadlamani Surya Prakash, Rajesh R Nair, Dharmesh Soneji, Sandeep Thareja, Pankaj P Rao, Jyotindu Debnath, Gurjeet Singh Chowdhary, Bhupesh Guleria, Neerja Kushwaha, Amit Biswas, Rama H, Vijay Bohra, Harshit Khurana, Sanjeevan Sharma, Kundan Mishra

**Affiliations:** 1 Department of Medical Oncology, Command Hospital, Lucknow, IND; 2 Department of Medicine, Command Hospital, Lucknow, IND; 3 Department of Surgery, Command Hospital, Lucknow, IND; 4 Department of Radiology, Command Hospital, Lucknow, IND; 5 Department of Medical Oncology, Command Hospital, Pune, IND; 6 Department of Transfusion Medicine, Command Hospital, Lucknow, IND; 7 Department of Lab Sciences and Molecular Medicine, Command Hospital, Lucknow, IND; 8 Department of Cardiology, Command Hospital, Lucknow, IND; 9 Department of Clinical Hematology and Bone Marrow Transplant (BMT), Command Hospital, Pune, IND; 10 Department of Clinical Hematology and Bone Marrow Transplant (BMT), Command Hospital, Lucknow, IND

**Keywords:** autologous stem cell transplantation (asct), investigator-initiated study, multiple myeloma (mm), platelet transfusion, romiplostim, thrombocytopenia

## Abstract

Background: There is no standard treatment to accelerate recovery from melphalan-induced thrombocytopenia in multiple myeloma (MM) patients undergoing autologous stem cell transplantation (ASCT). Romiplostim, a thrombopoietin receptor agonist, has been developed to upregulate platelet production.

Objective: This study aimed to assess the efficacy and safety of romiplostim in reducing platelet transfusions post-ASCT in MM patients.

Methods: This was an investigator-initiated, single-center, open-label, comparative pilot study conducted from January to December 2022. We evaluated 18 melphalan-conditioned MM patients treated with pegylated granulocyte colony-stimulating growth factor (GCSF) (6 mg subcutaneously, SC, on day 2) and romiplostim (250 mcg SC on day 3) post-ASCT (romiplostim cohort). We compared them with 56 MM patients who had undergone ASCT and received GCSF alone (conventional or retrospective cohort, enrolled from 2015 to 2021). Efficacy endpoints included the number of mean single donor platelet (SDP) and packed red blood cell (PRBC) transfusions, time to platelet and neutrophil engraftment, and safety and tolerability assessments.

Results: In the romiplostim cohort, the average number of SDP transfusions required was significantly lower than that in the conventional cohort (1.39 vs. 3.40, p = 0.0001). Platelet engraftment occurred significantly faster in the romiplostim cohort versus the conventional cohort (9.72 vs. 12.57 days, p = 0.0018). Neutrophil engraftment days and PRBC transfusion requirements were comparable between the two groups. No deaths or major safety concerns were reported.

Conclusion: Romiplostim demonstrated an improved efficacy and a favorable safety profile compared to the conventional approach in patients undergoing ASCT.

## Introduction

Multiple myeloma (MM) is a plasma cell neoplasm and is commonly treated with autologous stem cell transplantation (ASCT) following high-dose melphalan [1]. The primary goal of management is the eradication of residual disease and potential chemoresistance. However, the conditioning regimen used often leads to pancytopenia, with severe thrombocytopenia posing a significant concern due to the associated risks of bleeding, prolonged hospital stay, and an increased utilization of medical resources [1,2]. Currently, the management of conditioning regimen-related thrombocytopenia after ASCT involves the administration of platelet transfusions given during the peripheral blood count nadir to prevent bleeding events [3,4]. Platelets are a limited resource and are associated with short- and long-term complications, including transfusion-related reactions and induction of alloreactive platelet refractoriness, respectively [5]. Moreover, responses to platelet transfusions are variable and often transient in the early post-ASCT period, primarily because of factors that increase peripheral consumption, such as organ dysfunction, infections, and sepsis [6,7]. These challenges are further accentuated by logistic issues, lack of suitable blood donors and/or single donor recruitment, and public health emergencies like the COVID-19 pandemic, thus necessitating the exploration of alternative strategies to ensure standard-of-care therapy for patients during these periods.

Romiplostim, a thrombopoietin receptor agonist (TPO-RA), is approved by the United States Food and Drug Administration and the European Medicines Agency for treating patients with chronic immune thrombocytopenia (ITP) [8], and romiplostim biosimilar was approved by the Drugs Controller General of India in April 2019 for the management of chronic ITP. The success observed in the management of ITP prompted further exploration of romiplostim in the management of chemotherapy-induced thrombocytopenia (CIT) [9-11]. In a study by Soff et al., the administration of weekly romiplostim for CIT exhibited notable improvements and sustained platelet counts, effectively minimizing disruptions in chemotherapy with no major safety concerns [12]. Scordo et al. recently published an open-label pilot study, which evaluated the efficacy and safety of romiplostim for thrombocytopenia following ASCT [13] and demonstrated promising efficacy and safety post-ASCT.

There is a lack of data on the use of romiplostim in patients with MM undergoing ASCT, especially in emerging economies like India. Therefore, we conducted this single-center, investigator-initiated pilot study to evaluate the therapeutic potential of romiplostim in enhancing platelet recovery and reducing the need for platelet transfusion in MM patients undergoing ASCT.

## Materials and methods

Study design and patients

We conducted a prospective, single-center, investigator-initiated pilot study between January and December 2022 at Command Hospital, Lucknow, India, to assess the efficacy and safety of romiplostim in 18 MM patients (romiplostim cohort) aged ≥18 years old with adequate organ function and performance status, and undergoing planned ASCT. Patients with a history of symptomatic or incidental venous thromboembolism in the last six months before ASCT were included if they had been on and tolerated anticoagulants for over six months. The exclusion criteria included a prior diagnosis of myeloid malignancy, a symptomatic arterial thrombotic event in the last six months before ASCT, a diagnosis of ITP, or prior treatment with romiplostim or any other TPO-RA or any other investigational platelet-producing agents. The prospective data was compared with a retrospective cohort of 56 MM patients who had previously undergone ASCT at our center. The study was approved by the Institutional Review Board, and written informed consent was obtained from all participants.

Treatment and outcomes

Romiplostim/Study Cohort (Novel Approach)

**Figure 1:**
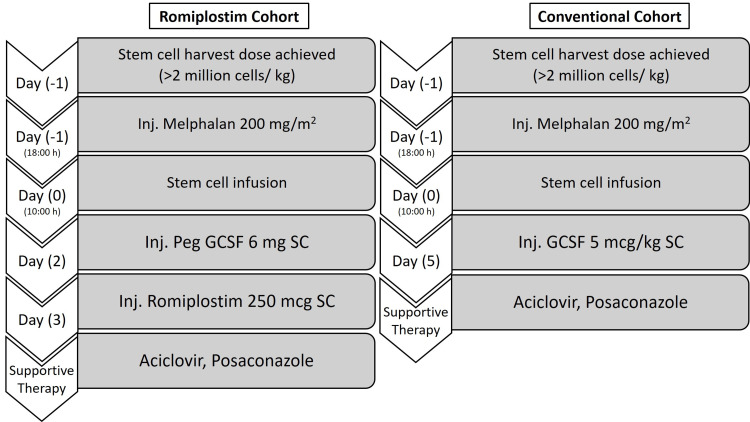
Study cohort Peg-GCSF: pegylated granulocyte colony-stimulating growth factor; SC: subcutaneously

Eligible MM patients received high-dose melphalan (conditioning regimen) and were prospectively followed up from the date of melphalan infusion to day +30 post-ASCT. Following stem cell infusion, a novel protocol was implemented, consisting of a single SC injection of pegylated granulocyte colony-stimulating growth factor (Peg-GCSF) 6 mg on day 2 and romiplostim 250 mcg SC (manufactured by Intas Pharmaceuticals Ltd., Ahmedabad, India) on day 3 (Figure 1). The primary efficacy endpoint was the mean number of SDP transfusions required. The secondary efficacy endpoints included the number of packed red blood cell (PRBC) transfusions required and the time to platelet and neutrophil engraftment. Prophylactic transfusion thresholds were set at 10,000/mm^3^ for platelets and 8 g/dL hemoglobin for RBC. Safety and tolerability were rigorously monitored throughout the study for adverse events (AEs).

*Control Cohort (Retrospective or Conventional Cohort*​​​​​​*)*

The control cohort, also known as the conventional cohort, consisted of 56 patients with MM who underwent ASCT between 2015 and 2021. The generated data was used to compare with that of the romiplostim cohort. The control cohort followed the conventional approach and received daily injections of GCSF 5 mcg/kg SC until neutrophil engraftment, without the use of romiplostim, from day 5 following stem cell infusion (Figure 1).

Stem-cell mobilization and harvesting

All patients underwent peripheral blood stem cell (PBSC) mobilization using GCSF at 10 mcg/kg/day, administered as a single dose for four consecutive days. If the peripheral blood CD34+ stem cell count was below 20 cells/µL on day 4, an injection of plerixafor (24 mg SC) was given the night of day 4, approximately 11 hours before harvesting PBSCs. PBSCs were harvested on day 5, with a targeted minimum yield of 2 × 10^6^ cells/kg, and stored overnight at 4°C. These were used for stem-cell transplantation.

Stem-cell transplantation

High-dose (200 mg/m^2^) melphalan was administered as a 10-minute infusion in the evening following the PBSC harvest. The harvested PBSCs were infused 16 hours after melphalan therapy. Laboratory investigations, including daily complete blood counts and a comprehensive metabolic panel, were monitored until engraftment. Standard supportive therapy included institutional protocols of acyclovir and posaconazole starting from day +1 of ASCT. Prophylactic SDP transfusions were administered for platelet counts <10,000/mm^3^ or in cases of clinical bleeding, while PRBC transfusions were administered for hemoglobin <8.0 g/dL.

Statistical analysis

The primary endpoint assessed the number of SDP transfusions required post-ASCT in both arms, while secondary endpoints included the days needed for platelet engraftment and PRBC requirements. An intention-to-treat analysis was conducted to assess efficacy outcomes. Mean and standard deviation were calculated and compared for continuous variables using the t-test. In contrast, categorical variables were analyzed using the chi-square test or Fisher's exact test as appropriate. Engraftment was evaluated using time-dependent analysis. All tests were two-sided, with a significance threshold set at p <0.05. AEs were assessed and graded weekly until seven days after the last romiplostim dose using the National Cancer Institute Common Terminology Criteria for Adverse Events version 5.0 (National Cancer Institute, Bethesda, MD).

## Results

Demographics and baseline characteristics

The study included 18 and 56 MM patients in the romiplostim and conventional cohorts, respectively, all of whom underwent ASCT. Both cohorts exhibited a male preponderance of 78%. The median age was 62 years (range 35-70) and 57 years (range 30-76) in the romiplostim and conventional cohorts, respectively (Table 1). The median PBSC dose transfused was 3.8 × 10^6^ cells/kg (range 1.23-10.5) in the romiplostim cohort and 3.67 × 10^6^ cells/kg (range 1.5-30.1) in the conventional cohort. The demographic and baseline characteristics were well balanced, and there were no significant differences between the two groups.

**Table 1:** Baseline demographics NA: not applicable; SD: standard deviation

Parameters	Novel cohort (n = 18)	Conventional cohort (n = 56)	p value
Gender distribution			
Male:female	14:4	44:12	NA
Age (years)			
Mean (SD)	59 (10.35)	56.78 (8.94)	0.3808
Median (range)	62 (35-70)	57 (30-76)	NA
Stem cell dose (million cells/kg)			
Mean (SD)	4.62 (2.64)	5.72 (4.97)	0.3731
Median (range)	3.8 (1.23-10.5)	3.67 (1.5-30.1)	NA
Melphalan dose (mg/m^2^)			
Mean (SD)	178.88 (26.98)	187.5 (20.02)	0.1463
Median (range)	200 (140-200)	200 (140-200)	NA

Efficacy and safety

**Figure 2:**
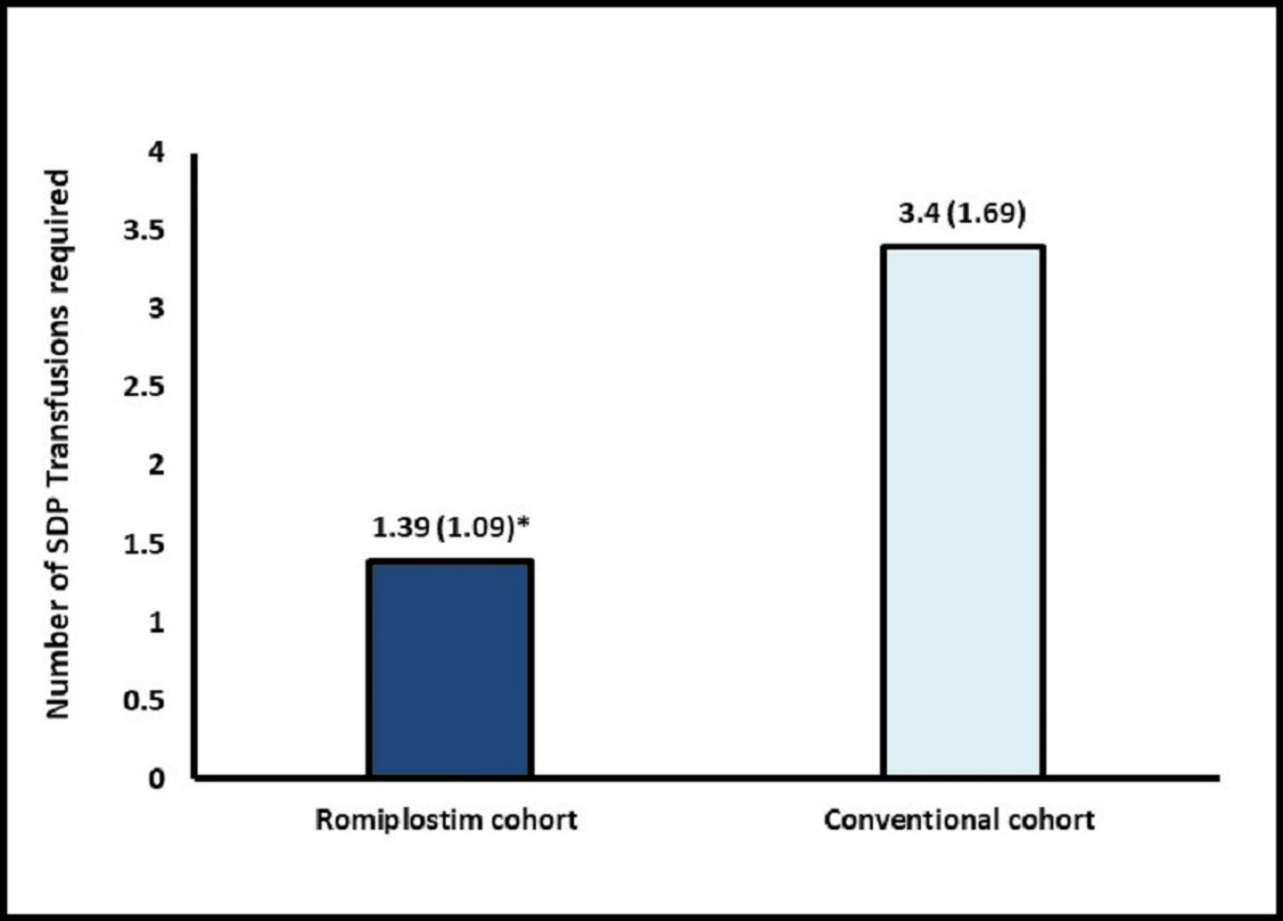
SDP transfusion rate ^*^p = 0.0001 SDP: single donor platelet

**Figure 3:**
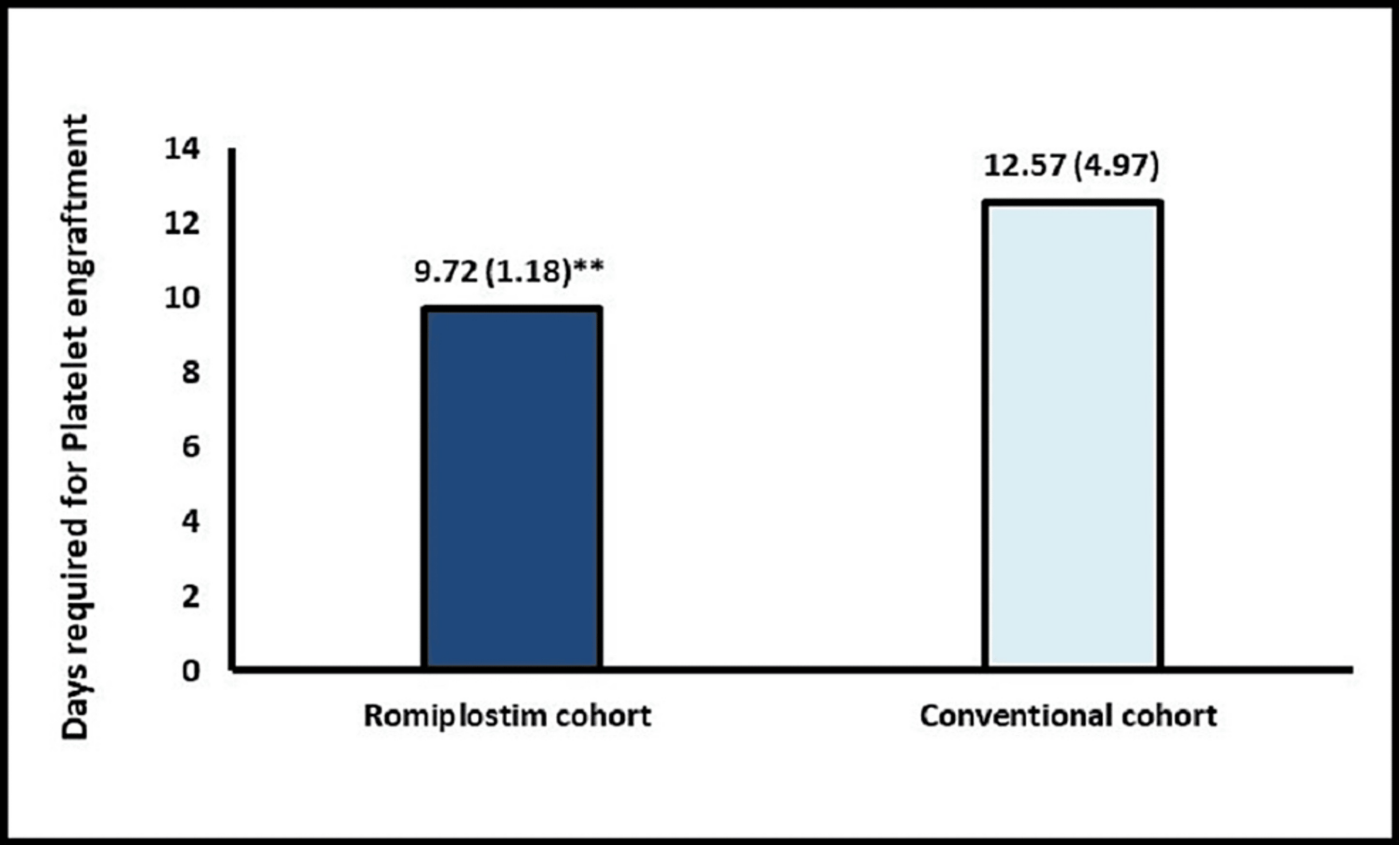
Platelet engraftment ^**^p = 0.0189

**Figure 4:**
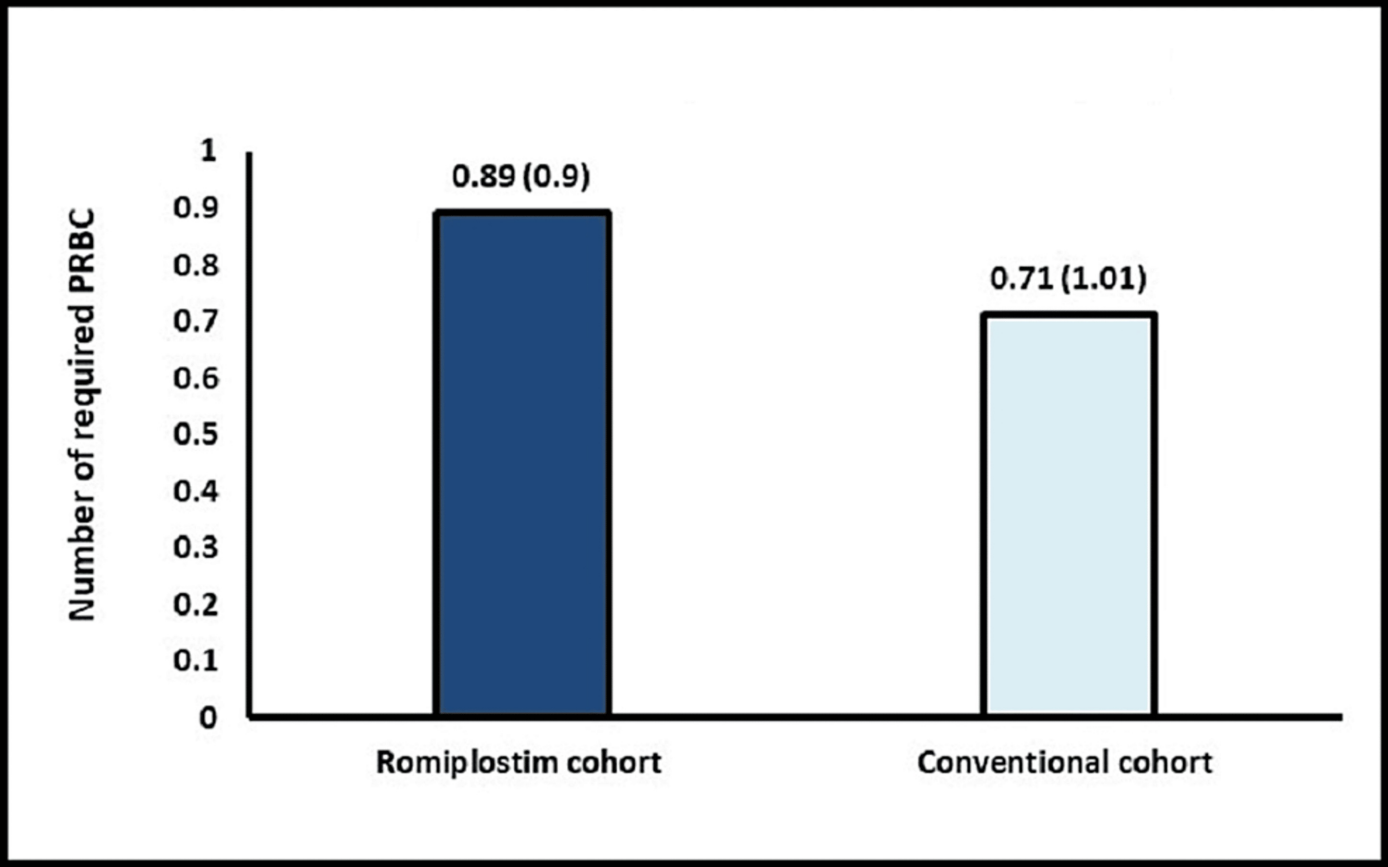
PRBC transfusion rate p = 0.5022 PRBC: packed red blood cell

The mean number of SDP transfusions was significantly lower in the romiplostim cohort vs. the conventional cohort (1.39 vs. 3.4, p = 0.0001) (Figure 2). The median time to platelet engraftment was significantly shorter in the romiplostim cohort at 9.72 vs. 12.57 days in the conventional cohort (p = 0.0189) (Figure 3). However, the median PRBC transfusion requirements were comparable between both groups (0.89 vs. 0.71, p = 0.5022)** **(Figure 4). The median time to neutrophil engraftment was approximately nine days in both cohorts. Importantly, all 18 patients in the romiplostim cohort achieved successful platelet and neutrophil engraftment. The posttransplant characteristics were similar between the two cohorts, with no bleeding complications observed during the post-ASCT period. The safety profile of the romiplostim cohort was favorable, with no major safety concerns reported, demonstrating that the novel approach of using romiplostim was safe and well tolerated in MM patients undergoing ASCT. Overall, these findings support the use of romiplostim as a potential treatment strategy for MM patients undergoing ASCT.

## Discussion

The present study aimed to evaluate the therapeutic potential of romiplostim in reducing platelet transfusion requirements and enhancing platelet engraftment in MM patients undergoing ASCT. Furthermore, romiplostim treatment at the studied doses appeared safe in these patients, without any notable increase in toxicities. Although we did not have absolute or relative platelet counts in the present or our retrospective study for comparison, we believe that the findings of this study are novel and may have clinical significance for the following reasons. Firstly, our study demonstrates a significant reduction in the mean number of SDP transfusions required in the romiplostim cohort compared to the conventional cohort. This reduction not only minimizes the risks associated with frequent transfusions, such as transfusion reactions and alloimmunization, but also alleviates the burden on blood bank resources in emerging economies like India, which is particularly critical in the context of global platelet shortages. Secondly, the shorter time to platelet engraftment observed in the romiplostim cohort suggests that romiplostim may enhance thrombopoietic recovery post-ASCT. A faster platelet engraftment can reduce the duration of thrombocytopenia, lowering the risk of bleeding complications and potentially shortening hospital stays [13]. This has significant implications for patient safety and healthcare costs, as prolonged hospitalization and managing bleeding episodes can be resource-intensive and expensive [3,4]. Thirdly, to the best of our knowledge, this approach has been utilized for the first time in patients undergoing ASCT in India. This pioneering strategy has the potential to make transplants more affordable and reduce acute and long-term risks associated with multiple transfusions. Finally, the favorable safety profile of romiplostim, with no major AEs reported in our study, highlights its potential as a safe adjunct to current post-ASCT supportive care protocols. This safety finding is crucial as it supports the integration of romiplostim into clinical practice without adding undue risk to patients. Together, these findings suggest that romiplostim could become a valuable therapeutic strategy in improving outcomes for MM patients undergoing ASCT.

Our study cohort was relatively homogeneous, as all patients received autologous grafts and identical supportive care. In patients who have undergone ASCT, studies involving TPO receptor agonists have mainly concentrated on enhancing thrombopoiesis in cases of prolonged thrombocytopenia post-allogeneic SCT. In contrast, we employed romiplostim along with GCSF proactively to reduce the need for platelet transfusion and the duration of neutropenia, respectively [14]. We devised a novel protocol to reduce the need for prophylactic transfusions based on the evidence supporting the use of TPO-RA (mainly romiplostim) in posttransplant settings. Coltoff et al. [15] utilized weekly dosing of romiplostim starting from day 2 of the transplant to accelerate platelet recovery. Al-Nawakil et al. [16] reported that romiplostim reduced the duration of severe thrombocytopenia from 4 to 2.6 days in 13 post-ASCT patients. In 2018, Al-Samkari et al. [17] demonstrated that romiplostim use in CIT was safe and led to rapid platelet recovery. They found that patients with baseline platelet counts <75,000/µL achieved platelet counts ≥75,000/µL within a median of seven days following a single dose of romiplostim. Given these findings, we designed our institutional protocol to minimize the need for prophylactic transfusions, acknowledging that transfusions carry risks such as acute reactions and potential infections [18]. The goal was to establish a more restrictive transfusion policy. Our novel approach involved a 6-mg SC injection of Peg-GCSF on day 2 and a 250-mcg SC injection of romiplostim on day 3. The results were promising and demonstrated a significant reduction in the mean number of SDP transfusions required in the romiplostim cohort compared to the conventional cohort (1.39 vs. 3.40, p = 0.0001). Furthermore, the median time to platelet engraftment was significantly shorter in the romiplostim cohort than in the conventional cohort (9.72 vs. 12.57, p = 0.0189). The time to neutrophil engraftment was similar between cohorts, with a median of approximately nine days. Importantly, all patients in the romiplostim cohort achieved successful platelet and neutrophil engraftment, and there were no significant posttransplant complications or major safety concerns, suggesting that romiplostim is a safe and effective intervention in this setting. This innovative strategy reduces the dependency on extrinsic blood products and mitigates the risks associated with frequent transfusions, making it a valuable approach for improving patient outcomes in MM undergoing ASCT.

The findings of our study are consistent with those of Scordo et al. [13], who conducted an open-label pilot study of romiplostim at a dose of 3 μg/kg SC once weekly for thrombocytopenia after ASCT in patients with MM and non-Hodgkin's lymphoma. In this study, the use of romiplostim was associated with improved platelet counts and reduced need for platelet transfusions, albeit with some delay in achieving the desired engraftment thresholds [13]. Our study corroborates the efficacy of romiplostim in reducing platelet transfusion requirements, but we observed a more favorable timeline for platelet engraftment. This difference might be attributed to variations in study design, patient populations, or romiplostim dosing schedules. Scordo et al. administered romiplostim weekly, whereas we administered a single dose of romiplostim on day 3 post-ASCT, which might have contributed to the differences in the time to platelet engraftment observed between the two studies [13]. The significant reduction in SDP transfusions and a shorter time to platelet engraftment observed in our study are clinically meaningful outcomes. Thrombocytopenia and the associated need for platelet transfusions present substantial challenges in the post-ASCT period, including increased risk of bleeding, prolonged hospitalization, and higher healthcare resource utilization [3,4]. By effectively reducing the number of platelet transfusions required, romiplostim can potentially mitigate these challenges, leading to improved patient outcomes and reduced healthcare costs. Moreover, our study's safety profile of romiplostim was favorable, with no major adverse events reported, such as venous thromboembolic events, beyond what is typically observed in patients following high-dose therapy and ASCT [19,20]. This finding is crucial as it suggests that romiplostim can be safely incorporated into post-ASCT management without adding undue risk to patients. The absence of significant safety concerns in our study aligns with the findings of Scordo et al. [13], who also reported no appreciable increase in the risk of thrombosis or adverse impacts on bone marrow function.

Despite the promising results, our study has several limitations. First, the sample size was small, comprising only 18 patients in the romiplostim cohort and 56 in the conventional cohort. Additionally, our study was conducted at a single center, which may limit the applicability of the findings to other settings with different patient demographics or clinical practices. The comparison with historical controls, although informative, is not as robust as a randomized controlled trial. A larger phase 3 trial would be particularly valuable in validating the role of romiplostim for managing thrombocytopenia in MM patients undergoing ASCT.

## Conclusions

In conclusion, the use of romiplostim in MM patients undergoing ASCT significantly reduce the need for platelet transfusions and shortens the time to platelet engraftment, with a favorable safety profile. These findings support the potential of romiplostim as an effective strategy for managing thrombocytopenia in the post-ASCT setting.

## References

[1] Wandt H, Schaefer-Eckart K, Wendelin K (2012). Therapeutic platelet transfusion versus routine prophylactic transfusion in patients with haematological malignancies: an open-label, multicentre, randomised study. Lancet.

[2] Stanworth SJ, Estcourt LJ, Powter G (2013). A no-prophylaxis platelet-transfusion strategy for hematologic cancers. N Engl J Med.

[3] Gajewski JL, Johnson VV, Sandler SG, Sayegh A, Klumpp TR (2008). A review of transfusion practice before, during, and after hematopoietic progenitor cell transplantation. Blood.

[4] Schiffer CA, Bohlke K, Delaney M (2018). Platelet transfusion for patients with cancer: American Society of Clinical Oncology Clinical Practice Guideline update. J Clin Oncol.

[5] Kaufman RM, Assmann SF, Triulzi DJ, Strauss RG, Ness P, Granger S, Slichter SJ (2015). Transfusion-related adverse events in the platelet dose study. Transfusion.

[6] Gordon B, Tarantolo S, Ruby E, Stephens L, Lynch J, Kessinger A, Haire W (1998). Increased platelet transfusion requirement is associated with multiple organ dysfunctions in patients undergoing hematopoietic stem cell transplantation. Bone Marrow Transplant.

[7] Claushuis TA, van Vught LA, Scicluna BP (2016). Thrombocytopenia is associated with a dysregulated host response in critically ill sepsis patients. Blood.

[8] (2024). NPLATE® (romiplostim) for injection, for subcutaneous use. Highlights of prescribing information. Approval.

[9] Parameswaran R, Lunning M, Mantha S, Devlin S, Hamilton A, Schwartz G, Soff G (2014). Romiplostim for management of chemotherapy-induced thrombocytopenia. Support Care Cancer.

[10] Al-Samkari H, Soff GA (2021). Clinical challenges and promising therapies for chemotherapy-induced thrombocytopenia. Expert Rev Hematol.

[11] Al-Samkari H, Parnes AD, Goodarzi K, Weitzman JI, Connors JM, Kuter DJ (2021). A multicenter study of romiplostim for chemotherapy-induced thrombocytopenia in solid tumors and hematologic malignancies. Haematologica.

[12] Soff GA, Miao Y, Bendheim G (2019). Romiplostim treatment of chemotherapy-induced thrombocytopenia. J Clin Oncol.

[13] Scordo M, Gilbert LJ, Hanley DM (2023). Open-label pilot study of romiplostim for thrombocytopenia after autologous hematopoietic cell transplantation. Blood Adv.

[14] Smith TJ, Bohlke K, Lyman GH (2015). Recommendations for the use of WBC growth factors: American Society of Clinical Oncology Clinical Practice Guideline update. J Clin Oncol.

[15] Coltoff A, Shreenivas A, Afshar S, Steinberg A (2019). A single-institution experience of performing bloodless transplant in Jehovah's Witness patients. Hematol Oncol Stem Cell Ther.

[16] Al-Nawakil C, Quarre MC, Heshmati F (2013). Autologous stem cell transplantation in patients who object to a blood transfusion: contribution of new pharmacological haematopoiesis support. Br J Haematol.

[17] Al-Samkari H, Marshall AL, Goodarzi K, Kuter DJ (2018). The use of romiplostim in treating chemotherapy-induced thrombocytopenia in patients with solid tumors. Haematologica.

[18] Ackfeld T, Schmutz T, Guechi Y, Le Terrier C (2022). Blood transfusion reactions-a comprehensive review of the literature including a Swiss perspective. J Clin Med.

[19] Chaturvedi S, Neff A, Nagler A, Savani U, Mohty M, Savani BN (2016). Venous thromboembolism in hematopoietic stem cell transplant recipients. Bone Marrow Transplant.

[20] Dahi PB, Lee J, Devlin SM (2021). Toxicities of high-dose chemotherapy and autologous hematopoietic cell transplantation in older patients with lymphoma. Blood Adv.

